# Node Collaborative Strategy for 3D Coverage Based on Hopping Adaptive Grey Wolf Optimizer in Wireless Sensor Network

**DOI:** 10.3390/s25247431

**Published:** 2025-12-06

**Authors:** Minghua Wang, Zhuowen Wu, Bo Fan, Yan Wang

**Affiliations:** 1 School of Electrical Engineering, University of South China, Hengyang 421001, China; mhwang@usc.edu.cn (M.W.); wuzhuowen1123@163.com (Z.W.); fanbohysd@163.com (B.F.); 2 Hunan Province Key Laboratory for Ultra-Fast Micro/Nano Technology and Advanced Laser Manufacture, University of South China, Hengyang 421001, China

**Keywords:** three-dimensional coverage, three-dimensional confident information coverage model, Hopping Adaptive Grey Wolf Optimizer, scheduling algorithm

## Abstract

Wireless sensor networks (WSNs) represent an emerging technology, among which coverage optimization remains a fundamental challenge. In specific application scenarios such as intelligent urban management, three-dimensional (3D) coverage models better reflect real-world requirements and thus hold greater research significance. To maximize the coverage performance of 3DWSNs, this study proposes a Three-Dimensional Confident Information Coverage (3DCIC) model based on the concept of multi-node cooperative information reconstruction, effectively extending the perceptual domain of sensor nodes. Furthermore, by incorporating adaptive dimension learning and opposition-based learning metchanisms into the wolf pack update strategy, we have developed the Hopping Adaptive Grey Wolf Optimizer (HAGWO) based on the GWO to optimize node deployment. Experimental results demonstrate the superior performance of the 3DCIC model, achieving coverage ranges 2.78 times, 4.41 times, and 4.00 times greater than those of conventional binary spherical models under regular tetrahedral, hexahedral, and octahedral node deployments, respectively. The proposed scheduling algorithm proves highly effective in both classical test functions and three-dimensional coverage problems.

## 1. Introduction

Wireless Sensor Networks (WSNs), as a pivotal enabling technology for the Internet of Things (IoT), have garnered significant attention in both industrial and academic circles [1]. WSNs is an autonomous, distributed network system composed of multiple sensor nodes that possess wireless communication and computational capabilities [2]. In recent years, WSNs have emerged as a dominant technology in both civilian and military domains, largely due to their convenient deployment, adaptable communication, affordability, and energy conservation [3]. As a result, WSNs have been widely applied in various fields, including military surveillance, ecological observation, intelligent urban management, disaster alert systems, extraterrestrial exploration, and industrial process control [4]. Moreover, in practical applications, WSNs are often susceptible to various environmental factors, making interference resistance a critical aspect of their design and performance [5,6].

It is widely acknowledged that the foundation of task execution in wireless sensor networks lies in effective monitoring of the area [7]. In addition to investigating the impact of wireless sensor energy consumption on the coverage area, enhancing the coverage range of sensor networks is of greater importance [8]. The rationality of node deployment is pivotal in enhancing coverage rates. Typically, the performance of deterministic deployment [9] often surpasses that of random deployment [10]; however, in practical scenarios, deterministic deployment is not a viable option, whereas random deployment is feasible. Therefore, optimizing the deployment scheme of sensor nodes to enhance the coverage of the monitored area is of paramount importance [11,12]. Furthermore, the node coverage model, which characterizes the sensing capabilities of nodes through their geometric relationships with spatial points, is central to the study of coverage issues in WSNs [13]. Different coverage models have a significant impact on the regional coverage rate. Therefore, constructing an excellent coverage model is key to enhancing regional coverage.

Additionally, scenarios for regional coverage are diverse. Compared to 2D scenarios, 3D practical application scenarios have a broad range of potential applications, making the study of 3DWSNs more critical [14]. The dimensionality of 3D coverage scenarios is higher and closer to reality, and it has become the main trend in current research. Consequently, devising a node deployment strategy that maximizes coverage in 3D environments while proposing a coverage model that closely reflects reality remains highly challenging. In UAV-assisted WSNs, three-dimensional deployment is particularly crucial [15].

Node deployment is inseparable from deployment strategies. Numerous heuristic algorithms, including Particle Swarm Optimization (PSO), Genetic Algorithm (GA), Ant Colony Optimization (ACO), and hybrid approaches such as PSO-GWO [16], have been employed in various studies to address multimodal and high-dimensional coverage optimization problems in WSNs [17]. However, these algorithms often require specific parameter tuning and are prone to premature convergence. The Grey Wolf Optimizer (GWO) has been widely adopted in diverse engineering applications due to its advantages of being free from specific parameters, having a simple structure, and exhibiting strong global search capabilities [18]. Nevertheless, when tackling certain multimodal and high-dimensional problems, the performance of GWO still requires further enhancement. For instance, In reference [19], Rodríguez et al. proposed a FGWO based on their research on fuzzy hierarchical. In reference [20], Dhargupta et al. introduced a selective opposition based grey wolf optimizer (SOGWO). Additionally, Nadimi-Shahraki et al. developed an improved grey wolf optimizer (IGWO) in reference [21]. These algorithmic variants and their enhancements have provided valuable directions for subsequent optimization research. Regarding node coverage models, the probabilistic sensing model [22] and binary sensing model [23] widely used in 3D scenarios, including their improved variants, only consider the sensing function of individual nodes in isolation and do not take into account that multiple nodes can enhance the sensing capabilities and quality of the target area through collaborative efforts.

In order to enhance the coverage rate of wireless sensor networks and improve the distribution of nodes, an improved GWO based on the hopping rate mechanism has been proposed, which further extends the applicability of the GWO algorithm in dimensional learning. Additionally, the three-dimensional confident information coverage (3DCIC) model has been introduced. This model leverages multiple sensor node pairs to estimate unsampled points, thereby significantly extending the effective coverage range of the nodes.

The main contributions of this paper are summarized as follows:

- The 3D cooperative node model based on field reconstruction theory, termed the 3DCIC model, is proposed for the first time. Under conditions of equal node numbers, it demonstrates multiple-fold gains in coverage range compared with conventional binary spherical models.
- Based on the hopping rate mechanism of opposition learning and adaptive dimension learning, Hopping Adaptive Grey Wolf Optimizer (HAGWO) algorithm is proposed, which enhances the search range and efficiency of wolves.
- A simulation platform was developed to assess the effectiveness of the proposed scheme. The experimental results indicate that, compared with existing model algorithms, the proposed HAGWO-3DCIC achieves favorable performance in terms of both regional coverage area and uniformity of node distribution.

This manuscript is structured into six parts. Section 2 offers a concise overview of prior research. Section 3 presents the CIC coverage model, formally defining the CIC coverage problem and its associated gain. The proposed methodology is elaborated in Section 4. Experimental results and analysis are provided in Section 5. Final remarks are summarized in Section 6.

## 2. Related Works

In 3DWSNs, numerous researchers have proposed optimization approaches from various perspectives. For instance, in reference [24], Barshandeh S et al. developed a localization algorithm for 3D IoTs, which effectively enhances the search efficiency of the network. In reference [25], Xu Y, Jiao W et al. proposed an energy-efficient routing protocol based on 3D WSNs, significantly extending the network lifespan. As the primary focus of this study, research on 3D coverage can be classified into two main categories: node deployment strategies and node sensing models.

### 2.1. 3D Node Deployment Strategies

Arivudainambi D et al. proposed a vertex coloring-based sensor deployment algorithm designed for 3D terrains, which effectively addresses coverage and connectivity requirements in IoT networks through structured graph-theoretic principles [26]. Hao Z et al. developed an improved PSO variant to optimize node deployment in 3D space, significantly improving overall network coverage performance while maintaining computational efficiency [27]. To overcome the challenges of node placement on complex 3D surfaces, Wang Z et al. presented an enhanced grey wolf optimizer (EGWO) that not only improves coverage quality but also reduces deployment costs through an efficient search mechanism [28]. Du Y proposed a hybrid methodology that integrates distributed PSO (DPSO) with a novel 3D Virtual Force (VF) model, achieving simultaneous optimization of both coverage and connectivity in complex 3D environments [29]. Yao Y et al. introduced an improved sparrow search algorithm (ISSA) specifically tailored for 3D node deployment, which demonstrates superior coverage performance through enhanced global search capabilities and convergence characteristics [30]. In reference [7], Yu et al. proposed an adaptive learning grey wolf optimizer (ALGWO) for coverage optimization in 3D regions. This approach, inspired by dimension learning in collective hunting behavior, incorporates an adaptive mechanism that effectively balances exploration and exploitation during the optimization process. Nevertheless, the algorithm still exhibits limitations in addressing high-dimensional coverage problems, particularly in maintaining population diversity and escaping local optima, indicating potential avenues for further improvement.

### 2.2. 3D Node Sensing Models

For node sensing models. In Article [31], Fu Xiao et al. first proposed abstracting the 3D coverage scenario into a sphere coverage problem and designed a coverage pattern based on rectangular prisms, which is essentially an extension of the binary model. Subsequently, in Article [32], Zhongsi Wang, Bang Wang et al. proposed an underwater three-dimensional Voronoi diagram coverage model. This model combines the structural characteristics of Voronoi diagrams with the z-axis movement characteristics of underwater anchors, thereby improving the coverage rate of the monitoring area, but it is limited to specific underwater environments. In Article [33], Saad et al. developed a real perception model based on the Bresenham line-of-sight, and in Article [34], Fu et al. proposed a node perception model based on DEM (Digital Elevation Model) data. Both of these models are based on probabilistic perception models and incorporate real monitoring scene data. However, they have not yet escaped the traditional probabilistic perception model framework.

In the aforementioned studies, although considerable efforts have been made to improve the three-dimensional node perception model, the focus has been solely on the sensing of individual nodes without considering the potential for collaboration among multiple nodes. Therefore, in this paper, based on an in-depth study and exploration of the spatial characteristics of environmental variable spaces, a collaborative coverage model for three-dimensional nodes is proposed. This model utilizes sensor nodes within the neighborhood of spatial points to reconstruct the information field, thereby expanding the coverage area. Since its fundamental principle involves using the data from the sensing data set of multiple sensor nodes in the network to reconstruct the information values of environmental attributes in the sensing target area, this model is referred to as the 3DCIC model in the following text.

## 3. The Proposed 3DCIC Model

### 3.1. The Principle of 3DCIC Model

The CIC model [35] is a node coverage model based on field reconstruction theory. We first construct a sensing scenario, assuming the need to monitor a physical phenomenon within the environment, such as air humidity in a forest. Sensor nodes are deployed at specific locations to sample the phenomenon, obtaining measured values at those points. Field reconstruction then uses these sampled values to interpolate (or estimate) the values of the phenomenon at unsampled spatial locations. Building upon existing field reconstruction theory, this paper proposes a 3DCIC model.

The physical phenomena in the environment typically exhibit spatiotemporal correlation characteristics. That is, to estimate the value of a physical property at a given spatial point, one must consider not only the sampled values at the current time but also those from previous moments. Let $z^{t}\left(xyz\right)$ denote the actual value of a spatial point (x,y,z) at time t, and ${\hat{z}}^{t}\left(xyz\right)$ represent its estimated value. The reconstruction function can be expressed as $f:\left\{z\left(s_{i}\right)|s_{i}\in S\right\}\to \hat{z}\left(xyz\right)\}$; here, *S* represents the set of sensor nodes, and the estimation function $\hat{z}\left(xyz\right)$ essentially represents a mapping from neighboring nodes to unsensed points within the domain. The objective of the field reconstruction function is to minimize the estimation error $|z^{t}\left(xyz\right)-{\hat{z}}^{t}\left(xyz\right)|$. Since the physical phenomenon in the environment constitutes a spatiotemporal stochastic process, $|z^{t}\left(xyz\right)-{\hat{z}}^{t}\left(xyz\right)|$ can be treated as a random variable with an unknown probability distribution. Therefore, we employ the root mean square error (RMSE) to evaluate the reconstruction quality at unsampled spatial points, yielding:

(1) $$\Phi \left(xyz\right)=\sqrt{\frac{1}{T}\sum_{t=1}^{T}{\left(z^{t}\left(xyz\right)-{\hat{z}}^{t}\left(xyz\right)\right)}^{2}},$$

Based on the aforementioned analysis, the definition of three-dimensional confident information coverage can be formally stated as follows.

Three-Dimensional Confident Information Coverage (Φ−Coverage): In a three-dimensional space with a specified reconstruction function *f*, a spatial point (x,y,z) achieves Φ-coverage status when the temporal average RMSE of its reconstructed information Φ(xyz) meets the criterion Φ(xyz)≤ε, where ε is the application-dependent threshold predetermined by network users. The complete three-dimensional space attains Φ-coverage if and only if all constituent spatial points satisfy this Φ-coverage condition.

The ordinary Kriging method is widely employed for field reconstruction [36,37,38], requiring only that the reconstructed physical phenomenon satisfies second-order stationarity. According to the definition of ordinary Kriging, it follows that:

(2) $${\hat{z}}^{t}\left(xyz\right)=\sum_{i=1}^{\left|S\right|}\lambda_{i}z^{t}\left(s_{i}\right),\;s_{i}\in S$$

Here, $\lambda_{i}$ represents the interpolation weights. Based on the unbiasedness condition of ordinary Kriging, we have $\sum_{i=1}^{n}\lambda_{i}$ = 1. The optimal weights are determined by minimizing the Kriging variance. By introducing a Lagrange multiplier μ, we construct a linear Kriging system consisting of n + 1 equations with n + 1 unknowns, expressed as:

(3) $$\left(\begin{array}{c} \lambda_{1}\\ \vdots \\ \lambda_{n}\\ \mu \end{array}\right)={\left(\begin{array}{cccc} \gamma (s_{1},s_{1}) & \cdots & \gamma (s_{1},s_{n}) & 1\\ \vdots & \ddots & \vdots & \vdots \\ \gamma (s_{n},s_{1}) & \cdots & \gamma (s_{n},s_{n}) & 1\\ 1 & \cdots & 1 & 0 \end{array}\right)}^{-1}\left(\begin{array}{c} \gamma (s_{1},p)\\ \vdots \\ \gamma (s_{n},p)\\ 1 \end{array}\right),$$

The above n = |S|, $\gamma (s_{i},p)$ and $\gamma (s_{i},s_{j})$ are the variogram of the monitored physical phenomenon, p(x,y,z). A set of optimal weight coefficients is obtained by Equation (3), After some algebraic operations, Φ(xyz) can be calculated as follows:

(4) $$\Phi \left(p\right)=\sqrt{\sum_{i=1}^{n}\lambda_{i}\gamma \left(s_{i},p\right)+\mu },$$

In spatial statistics, the variogram is a fundamental function that quantifies the spatial dependence of physical phenomena [38]. For a stationary process, the variogram can be expressed as a function of the Euclidean distance between two spatial points, such that γ(a,b) = γ(h), where h = d(a,b) represents the Euclidean distance between points a and b. Following [35], we employ the standard Gaussian variogram model to characterize the spatial physical phenomenon, given by:

(5) $$\gamma \left(h\right)=\left\{\begin{array}{cc} 0, & \mathrm{if}\;h=0\\ C_{0}+C\left(1-e^{-\frac{h^{2}}{a^{2}}}\right), & \mathrm{if}\;h>0 \end{array}\right.,$$

The constant a is related to the correlation range *D*, $D=\sqrt{3}a$. It should be noted that only points within the correlation range are considered spatially dependent. Consequently, the reconstruction domain for point p(x,y,z) is effectively a spherical region centered at p(x,y,z) with radius D.

### 3.2. Coverage Problem of 3DCIC Model

In a three-dimensional area, the monitoring region is divided into X×Y×Z net points. We assume *N* identical sensor nodes $S=\{s_{1},s_{2},\ldots ,s_{N}\}$ are deployed in the area, each with a communication radius *R* and a sensing radius $R_{s}$. According to the distribution of the nodes, they are divided into several sets, where nodes within the same set share sensing information. They collaborate based on Equation (4) and a pre-determined ε to sense the area.

It is commonly believed that if sensor nodes can cover *n* points within the three-dimensional area, the coverage rate is $\frac{n}{X\times Y\times Z}$. Thus, the coverage rate of the sensing area is defined as the proportion of grid points covered by the sensor nodes relative to the total number of grid points within the area. Let *X*, *Y*, and *Z* represent the coordinate dimensions along the x-axis, y-axis, and z-axis, respectively. The objective function for the coverage optimization of the three-dimensional sensing area is formulated as follows:

(6) $$OF_{3D}=\frac{\sum_{p\in X\times Y\times Z}C\left(S,p\right)}{X\times Y\times Z},$$

where C(S,p) represents a space point that can satisfy the node sensing condition.

**Figure 1:**
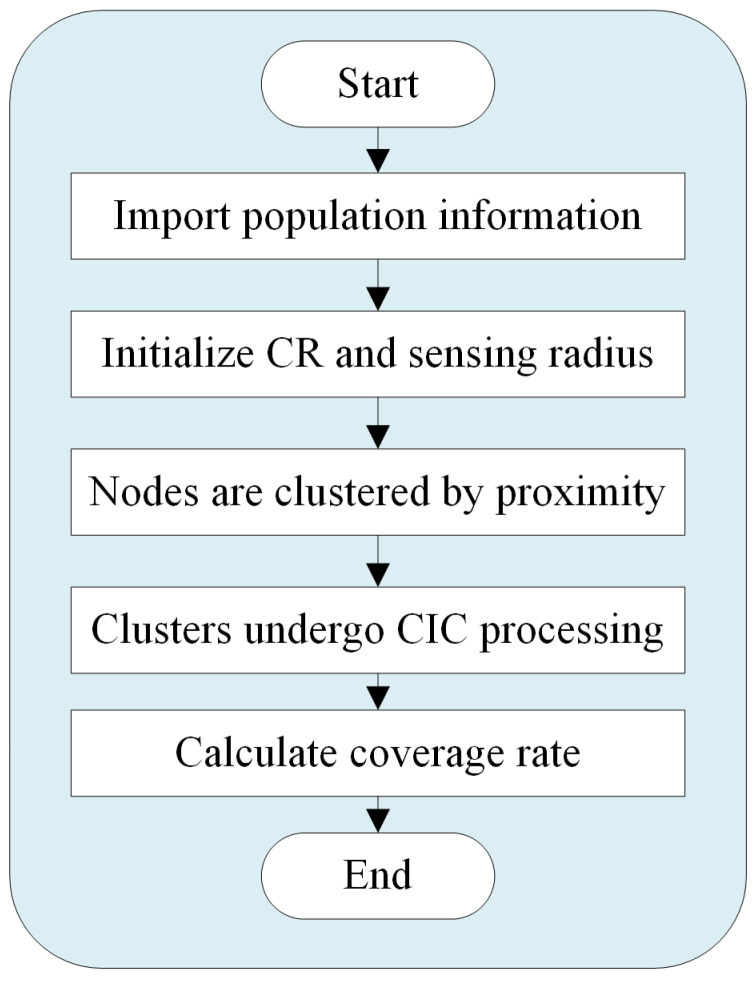
Flow chart of 3DCIC coverage optimization.

In a three-dimensional scenario, a metaheuristic algorithm is employed where each individual in the population is projected onto the x, y, and z axes. The dimensionality of the individuals within the population represents the number of nodes, with the node coordinates signifying the dimensional information. Figure 1 illustrates the coverage optimization process of the 3DCIC model.

### 3.3. Demonstration of Coverage Gain of 3DCIC Model

Generally speaking, node deployment can be categorized into two primary paradigms: deterministic deployment (also known as regular deployment) and stochastic deployment.

In two-dimensional deterministic deployment schemes, regular polygons are typically employed as the geometric topology for sensor node placement. For three-dimensional space, regular polyhedrons are generally adopted. This section utilizes common regular polyhedrons—tetrahedrons, hexahedrons (cubes), and octahedrons—as the topological shapes for node deployment, with nodes usually positioned at the vertices of these polyhedrons. If all spatial points within a polyhedron can be reliably covered by sensor nodes deployed at its vertices, the entire random field can be fully and reliably covered using a similar deployment pattern and geometric configuration.

In this section, to fully demonstrate the advantages of the 3DCIC model, deterministic deployment is adopted in simulations, with all node attributes kept identical and the RMSE threshold set to 0.5. In the simulation experiments, when a spatial coordinate is sensed, it is highlighted in yellow. It is assumed that if a spatial coordinate is sensed, its neighboring region is also effectively covered. In three-dimensional space, the coverage area of the binary model is a spherical region. To ensure no coverage gaps in regular polyhedron deployments, the radius of the polyhedron’s circumscribed sphere must equal the sensing radius of the sensor nodes. When deploying regular polyhedrons using the 3DCIC model, the maximum edge length is achieved by satisfying the condition Φ(0,0,0)<ε. For this study, the origin of the coordinate system is selected as the center of the polyhedron.

Based on the above analysis, the coverage gain of the 3DCIC model compared to the binary model is evaluated under different regular polyhedron deployment schemes.

**Figure 2:**
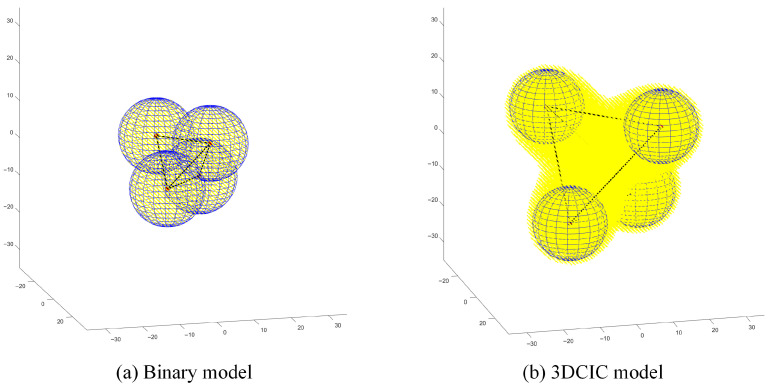
Comparison between binary model and 3DCIC model under regular tetrahedral deployment.

**Figure 3:**
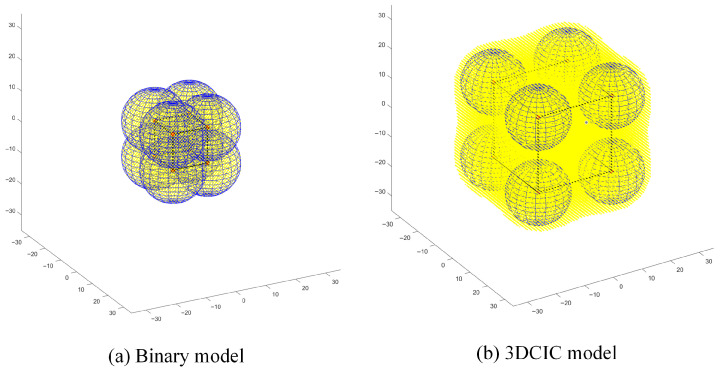
Comparison between binary model and 3DCIC model under regular hexahedral deployment.

**Figure 4:**
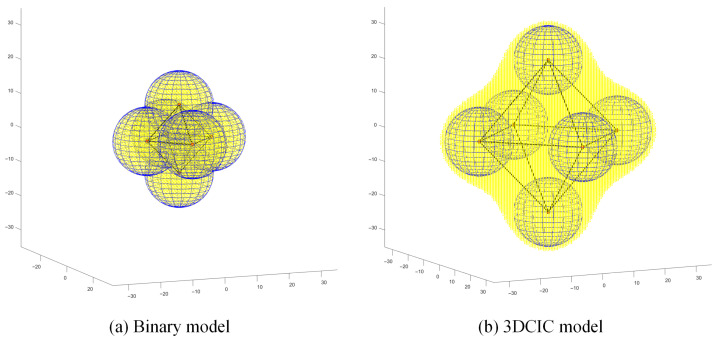
Comparison between binary model and 3DCIC model under regular octahedral deployment.

Figure 2 demonstrates that the 3DCIC model provides 2.78 times greater coverage area compared to the binary model in regular tetrahedral deployment scenarios, with statistically significant improvement. Regular hexahedral (cubic) deployment is relatively straightforward and represents one of the most commonly used approaches in three-dimensional deployment scenarios. As observed in Figure 3a, the limitations of traditional spherical deployment models become evident: to ensure complete coverage without voids, nodes often require dense placement, leading to significant resource redundancy. In contrast, Figure 3b demonstrates that under the same cubic deployment configuration, the 3DCIC model achieves substantially larger coverage areas compared to spherical deployment. Simulation results indicate that the 3DCIC model provides 4.41 times the coverage area of the binary model. Under regular octahedral deployment, Figure 4 demonstrates that when nodes are placed at each vertex of the octahedron, the 3DCIC model achieves significantly larger coverage compared to the binary spherical model. Quantitative analysis shows the 3DCIC model provides 4.00 times the coverage area of the binary model.

In deterministic deployment schemes, the 3DCIC model demonstrates significant advantages, achieving higher coverage rates with lower node density while maintaining the same number of nodes. Table 1 presents the magnification times of coverage range for the 3DCIC model compared to the binary model under different deployment configurations. Experimental results indicate that both regular hexahedral and octahedral structures exhibit superior performance in terms of coverage rate and resource utilization efficiency. In contrast, the regular tetrahedral structure shows greater spatial deployment flexibility under sparse node distribution conditions.

## 4. The Optimized HAGWO Algorithm

### 4.1. GWO

**Figure 5:**
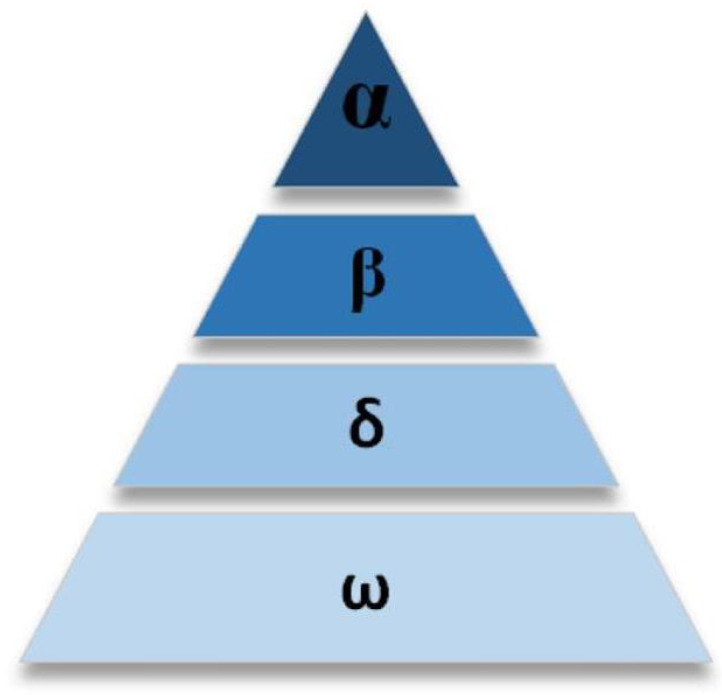
The social class of wolves.

The GWO [39] mimics the hunting process of wolf packs to solve problems, as shown in Figure 5, wolves α, β, and δ occupy the top three positions in the hierarchy, dominating the hunting activities of the wolf pack. The majority of the wolves are classified as ω, following the commands of the leading wolves. The process of updating the wolf pack’s positions can be divided into two phases: encircling and hunting. The encircling phase can be abstracted as follows:

(7) $$D=\left|C\cdot X_{p}\left(t\right)-X\left(t\right)\right|,$$

(8) $$X\left(t+1\right)=X_{p}\left(t\right)-A\cdot D,$$

where $X_{p}\left(t\right)$ is the location of the prey, X(t) is the location of the wolf at time *t*, and X(t+1) represents the location of the wolf at the next time step.

The control parameters *A* and *C* are calculated according to Equations (9)–(11).

(9) $$\begin{array}{cc} A & =2a\times r-a, \end{array}$$

(10) $$\begin{array}{cc} a & =2-2\times \frac{\mathrm{iter}}{\mathrm{Maxiter}}, \end{array}$$

(11) $$\begin{array}{cc} C & =2\times r, \end{array}$$

Here, *r* denotes a random number between 0 and 1, and *a* decreases from 2 to 0 as the number of iterations increases. After encircling the prey, the next phase is the hunting process, which is led by the top three wolves, as shown below:

(12) $$\begin{array}{cc} D_{\alpha } & =|C_{1}\cdot X_{\alpha }-X|,\\ D_{\beta } & =|C_{2}\cdot X_{\beta }-X|,\\ D_{\delta } & =|C_{3}\cdot X_{\delta }-X|, \end{array}$$

(13) $$\begin{array}{cc} X_{1} & =X_{\alpha }-A_{1}\cdot D_{\alpha },\\ X_{2} & =X_{\beta }-A_{2}\cdot D_{\beta },\\ X_{3} & =X_{\delta }-A_{3}\cdot D_{\delta }, \end{array}$$

(14) $$X\left(t+1\right)=\frac{X_{1}+X_{2}+X_{3}}{3}.$$

### 4.2. Opposition-Based Learning Mechanism

Opposition-Based Learning (OBL) is a machine learning method used in optimization algorithms within the field of computational intelligence, first introduced by Tizhoosh in 2005 [40]. The core concept of this strategy is to consider both the candidate solutions and their opposite solutions during the search process. Experiments have shown that, in the absence of prior knowledge about the optimization problem, opposite candidate solutions are more likely to reach the global optimum than random solutions. When GWO solves the problem, the initialized population is completely random, but different populations often affect the final result, so it is a trend to initialize the population with OBL. In [41], the authors discovered that, in addition to initializing the population, the hopping rate characteristic of OBL can also balance the exploration and exploitation of the wolf pack. The OBL can be represented by Equation (15): 

(15) $$X^{o}=Lb+Ub-X,$$

where $X^{o}$ is the inverse solution of *X*, Lb and Ub are the lower and upper bounds of the search space, respectively.

### 4.3. Hopping Adaptive Grey Wolf Optimizer

Dimension learning is an efficient search strategy. In references [7,21], a neighborhood is created for each wolf, within which there is information from other dimensions available for individual learning. The advantage of this method lies in its ability to expand the search range of the wolf pack, preventing it from becoming trapped in a local optimum during the later stages of iteration. Generally speaking, when the value of *a* is between 1 and 2, the wolf pack is in the exploration phase, during which the pack will endeavor to maximize its search scope. When the value of *a* is between 0 and 1, the wolf pack enters the exploitation phase, where it focuses on finding the optimal solution. Additionally, if the outcomes from the exploration phase are not favorable, the likelihood of the wolf pack becoming trapped in a local optimum during the exploitation phase increases.

Next, I will introduce the HAGWO based on the Adaptive Dimension Learning (ADL) mechanism [7] and the OBL jump mechanism. The proposed algorithm employs Dynamic Opposition Learning (DOL) [42] during the initialization phase, as DOL possesses asymmetric characteristics that effectively mitigate the risk of becoming trapped in local optima. By integrating ADL with OBL for updating the positions of the wolf pack, the algorithm is capable of expanding the search range while considering neighborhood dimension learning, thus balancing exploration and exploitation. The details of HAGWO are provided in Algorithm 2.

**Figure 6:**
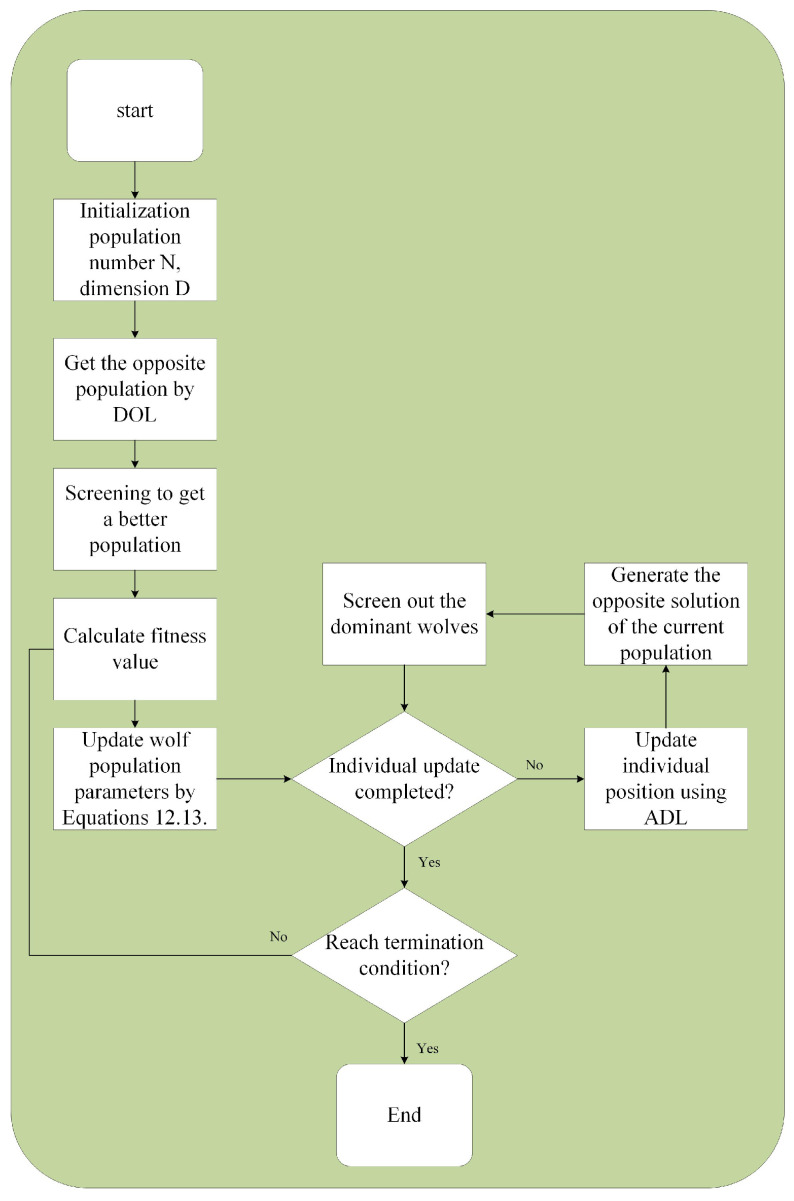
Flow chart of HAGWO.

The following is an analysis of the algorithm’s computational complexity. Assume the population size is *N*, the dimension of the problem set is *D*, and the maximum number of iterations is *T*, *O* represents the computational complexity of the algorithm. This analysis integrates the pseudo-code of Algorithms 1 and 2, along with the flowchart in Figure 6. The computational complexity of the standard GWO is O(N·D·T). The complexity of initialization using DOL is $O\left(N\cdot D\right)+O\left(N\cdot \log_{2}N\right)$. When using the ADL and OBL mechanisms for pack selection, the complexity is $O(N\cdot \left(D+N\cdot D+N\cdot \log_{2}N\right))$. Retaining the highest term, the complexity is $O(N^{2}\cdot D)$. Therefore, under the same number of iterations, the computational complexity of the HAGWO is $O(N^{2}\cdot D\cdot T)$. Compared to GWO, HAGWO has a higher algorithmic complexity, and the computation time for processing the same problem will also increase.


**Algorithm 1** GWO**Input:** Objective function *f*, search space dimension *D*, population size *N*, maximum number of iterations MaxIter
1. Initialize the grey wolf population *P*2. Calculate the fitness value of each individual3. Set the alpha, beta, and delta wolves as the three individuals with the highest fitness in the population4. For iter=2 to MaxIter5. Update the positions of the wolf pack using formulas (12) to (14)6. Calculate the fitness value of each wolf7. Update the α, β, and δ wolvesEnd for**Output:** The fitness value of the α wolf




**Algorithm 2** HAGWO **Input:** Objective function *f*, search space dimension *D*, population size *N*,maximum number of iterations Maxiter1. Initialize the grey wolf population *P*2. For i=1 to *N* do3. Generate inverse solutions using DOL4. End for5. Check boundaries6. Select the most suitable population7. Calculate the fitness value for each individual8. Set the alpha, beta, and delta wolves as the three individuals with thehighest fitness in the population9. For iter=2 to Maxiter do10. For i=1 to *N* do11. Update the positions of the wolf pack using Equations (12) to (14)12. For j=1 to *D* do13. The wolf pack performs adaptive dimension learning through ADL14. End for15. If rand < 0.4 then16. Generate inverse solutions for the current population using Equation (15)17. End if18. Select a more suitable population *P*19. End for20. End for**Output:** The fitness value of the α wolf


## 5. Performance Evaluation

In this chapter, all comparison algorithms and simulation experiments in this paper were implemented in MATLAB R2019a and executed on a Windows 11 system with a PC equipped with 16GB of memory and a 2.60 GHz i5-13500H processor.

The coverage performance of the HAGWO algorithm in a three-dimensional scenario is first verified through simulations using the classical binary model. Subsequently, the 3DCIC model is coupled with the HAGWO algorithm, ultimately resulting in an increase in coverage gain.

**Figure 7:**
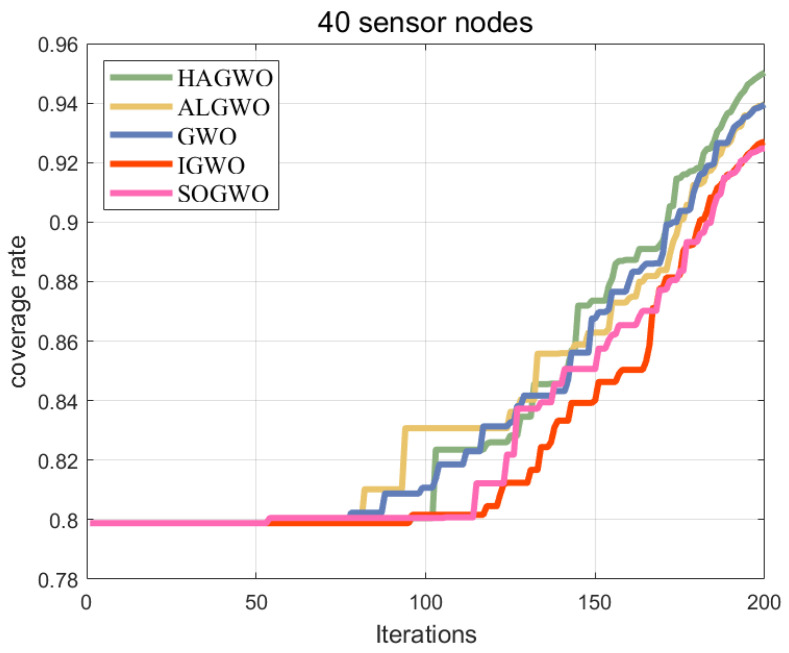
Coverage comparison of algorithms in a 3D obstacle-free scenario.

Based on the binary model as the node sensing model, the parameter settings refer to Table 2, and the optimization results are shown in Figure 7. After the iteration is completed, the coverage rate of HAGWO is 95.2%, ALGWO is 94.0%, GWO is 93.9%, IGWO is 92.7%, and SOGWO is 92.5%. The HAGWO algorithm demonstrates certain advantages over its competitors in optimizing coverage within 3D obstacle-free areas.

This study focuses on the sensing capability of nodes in WSNs. As energy consumption primarily results from node movement, it is approximated by the total distance traveled by the nodes [43,44]. After initial random deployment, the algorithm guides the nodes to their optimal positions, a process that accounts for the energy expenditure. The distance referred to here is the Euclidean distance between two points. A shorter average moving distance of the nodes corresponds to lower energy consumption. An analysis of energy consumption will be presented next, with all experimental parameters provided in Table 2. For this type of position-matching problem, the Hungarian algorithm [45] can be applied to ensure the shortest total moving distance. In Table 3, it can be unequivocally observed that the nodes scheduled by HAGWO exhibit the lowest average moving distance, which corresponds to minimal energy consumption.

**Figure 8:**
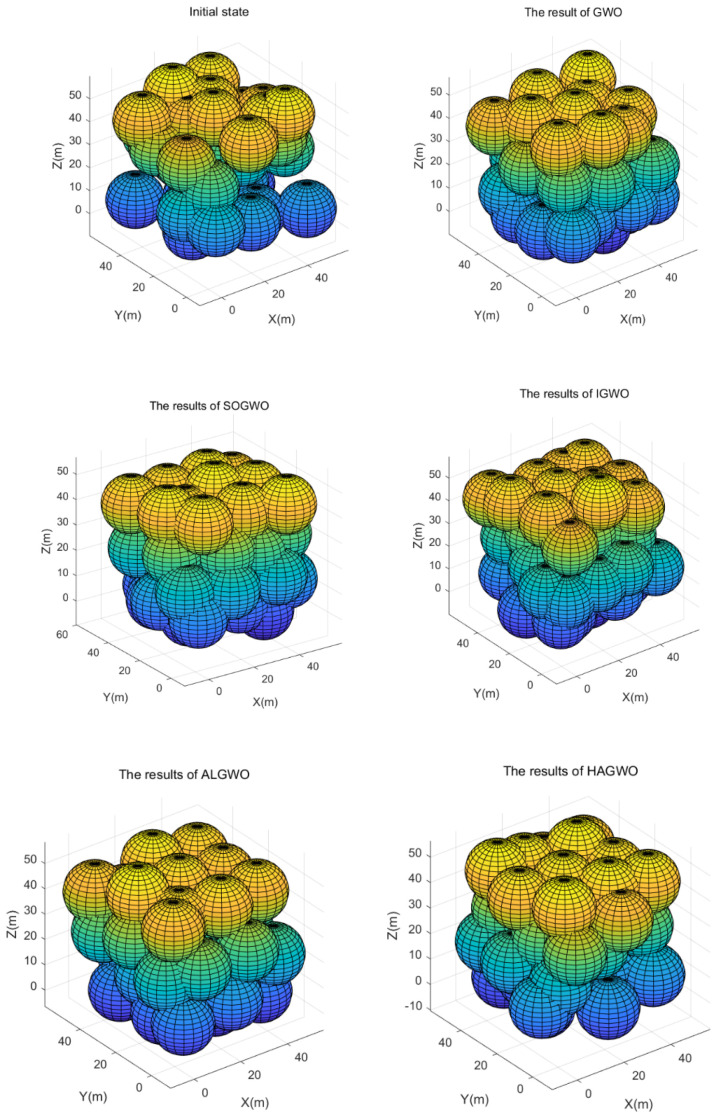
Distribution results of different node scheduling algorithms.

In Figure 8, it can be seen that in the 3D obstacle-free scenario, the initial state of random deployment has the characteristics of uneven node distribution and high overlap rate, which leads to the coverage rate of random deployment failing to meet the requirements of network users. Compared with the initial state, after using GWO for node scheduling, the nodes are obviously more dispersed and the node redundancy is reduced. In the node deployment strategy provided by SOGWO, the node distribution is more uniform. In comparison, IGWO provides a higher redundancy and lower coverage rate than GWO in the three-dimensional scenario. Both ALGWO and HAGWO provide good node distribution strategies with lower overlap rates and more balanced node distribution within the area. However, in terms of area coverage performance, HAGWO has better performance. Overall, HAGWO demonstrates superior coverage performance compared to the other four technologies, highlighting its effectiveness in wireless sensor network coverage optimization.

**Figure 9:**
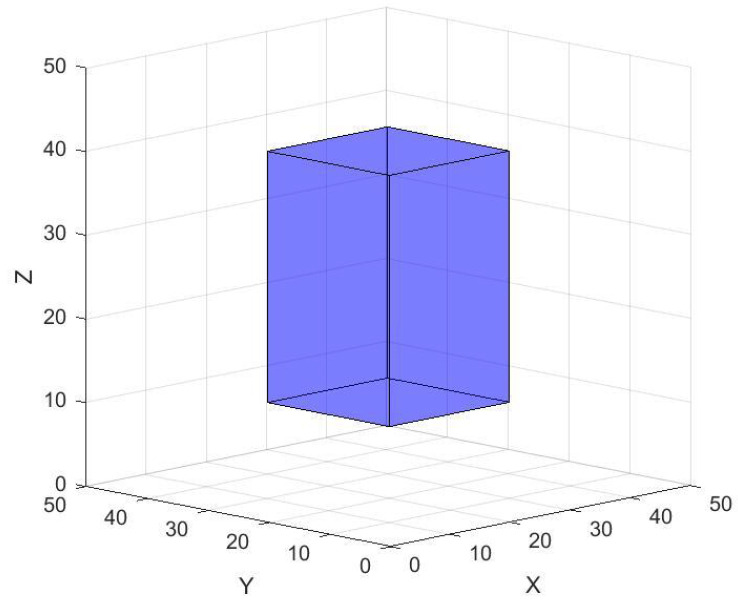
Cuboid obstacle in 3D scenario.

Meanwhile, scenarios involving obstacles are frequently encountered in real-world applications. To evaluate the effectiveness of the algorithm, a cuboid obstacle will be incorporated based on the network parameters defined in Table 2. This setup will subsequently be used to validate the algorithm’s coverage performance in obstructed environments. The cuboid obstacle is illustrated in Figure 9.

In this experiment, the node obstacle avoidance rule was adapted from reference [46] with modifications for the 3D scenario. When a node *a* is located inside an obstacle, its position requires correction. A line segment ac, perpendicular to the obstacle surface and twice the length of the node’s sensing radius, is defined originating from point *a*. The updated position lies along this segment. This process can be formulated as $X_{s}=f⊗ac$, where $X_{s}$ denotes the updated position, *f* is a randomly generated number within the interval [0, 1], and the symbol in the middle represents a geometric random interpolation operation. Given the reduced effective area in this obstacle-inclusive scenario, the number of nodes was set to 35.

**Figure 10:**
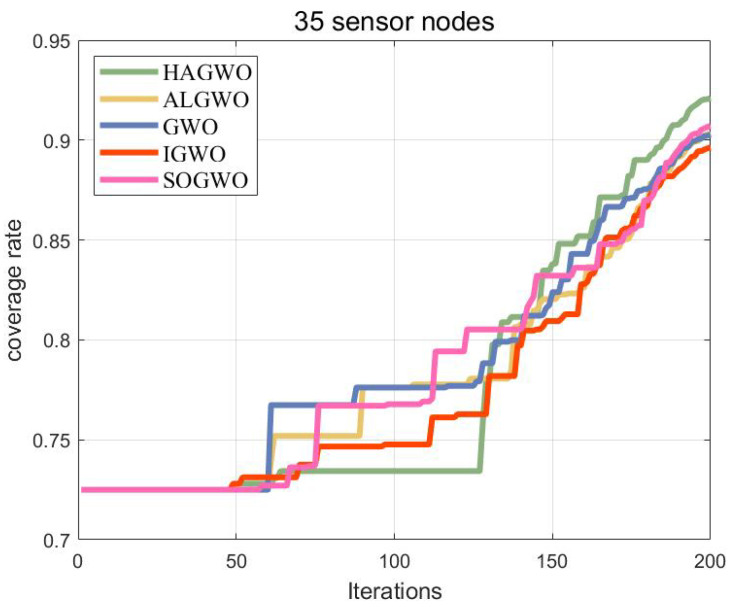
Coverage comparison of algorithms in a 3D obstacle scenario.

Figure 10 presents a comparison of the coverage rates achieved by different algorithms in the 3D obstacle scenario. The recorded coverage rates are 92.1% for HAGWO, 90.3% for ALGWO, 90.2% for GWO, 89.6% for IGWO, and 90.7% for SOGWO. It is evident that HAGWO maintains its effectiveness even in the presence of obstacles.

The above experiments are based on the classic binary model for the coverage performance test of HAGWO. In the following experiments, the performance of node deployment strategy based on random deployment will be evaluated using the 3DCIC model.

Conclusions drawn from simulations indicate that the 3DCIC model exhibits more pronounced gains as the number of nodes decreases. In the following discussion, we employ the HAGWO algorithm; other node scheduling algorithms are also expected to yield significant gains in theory, and interested readers are encouraged to conduct their own experiments. In this experiment, an additional comparison group using ALGWO in the binary model was incorporated to further highlight the coverage gain of HAGWO-3DCIC. Except for the variation in the number of nodes, all experimental parameters remain consistent with those listed in Table 2.

**Figure 11:**
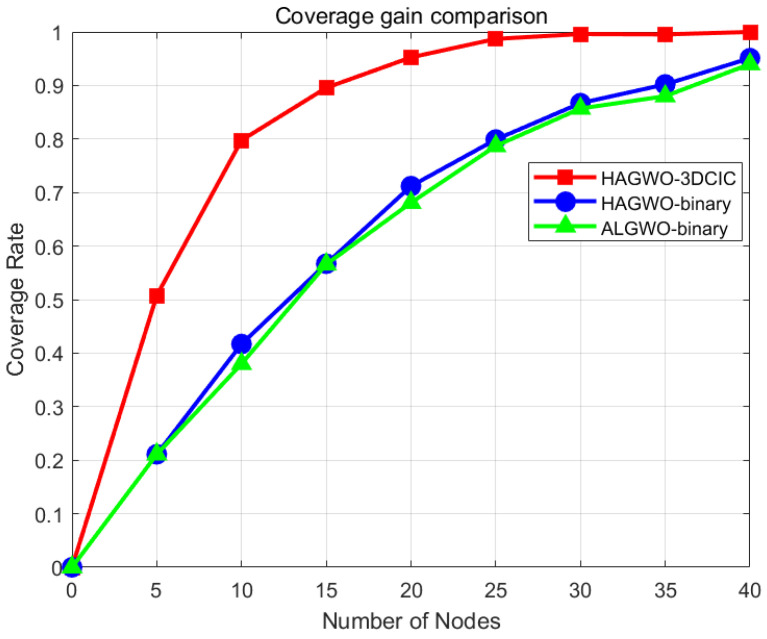
Coverage gain comparison between the 3DCIC and binary models with varying node counts in an obstacle-free scenario.

**Figure 12:**
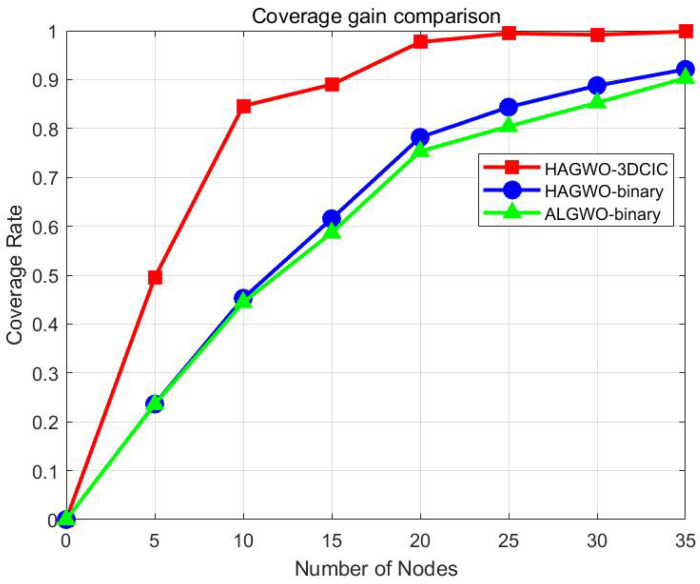
Coverage gain comparison between the 3DCIC and binary models with varying node counts in an obstacle scenario.

By observing Figure 11, it is evident that the 3DCIC model achieves the maximum gain relative to the binary model when the number of nodes in the area is 10, reaching a gain of 38%. Subsequently, as the number of nodes increases, the gain gradually decreases. When the number of nodes is 25, the coverage rate of HAGWO-3DCIC approaches 99%. Further increasing the number of nodes results in the coverage rate increasingly approaching 1, and thus the gain compared to other models becomes progressively smaller. Similarly, it can be observed from Figure 12 that HAGWO-3DCIC maintains a significant gain in an obstacle scenario.

**Figure 13:**
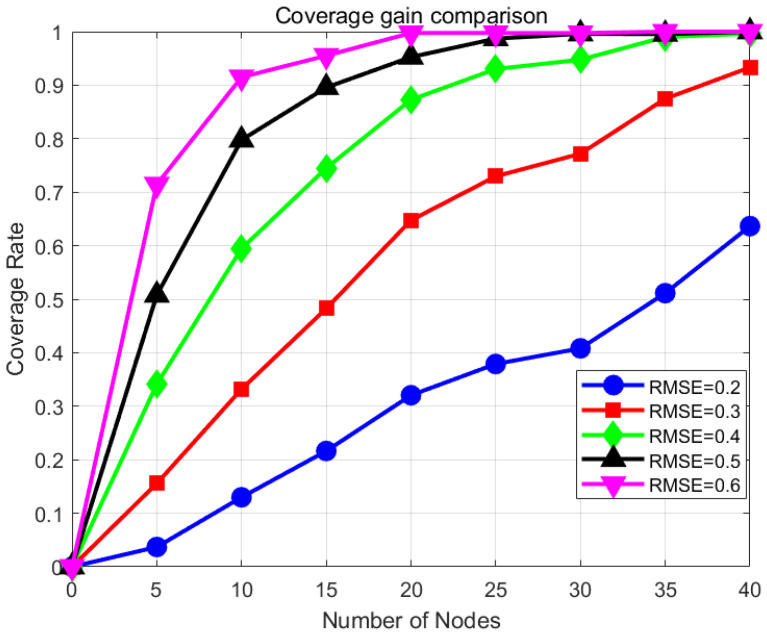
Influence of RMSE threshold on coverage rate under different nodes.

At the same time, different threshold values will also affect the sensing capability of 3DCIC. Theoretically, the smaller the RMSE threshold value ε, the more stringent the network users’ requirements for coverage quality, and the corresponding coverage rate will be lower. In this experiment, the scheduling algorithm also uses HAGWO, other network parameters besides variables are listed in Table 2. In Figure 13, it is evident that the coverage rate increases with the increase of the threshold ε, which is as expected. Moreover, when the value of ε is less than 0.3, the performance of HAGWO-3DCIC is not as good as the binary model. Therefore, too high a requirement for coverage quality from network users can also lead to a decrease in the advantages of the 3DCIC model. It is recommended that the value of ε be in the range of [0.4, 0.6].

The primary objective of this study is to investigate network coverage capability. Therefore, an idealized environment was adopted in the simulation setup, with simplifications applied to real-world interference factors. Furthermore, since the research emphasizes the sensing performance of nodes, an ideal communication model was temporarily employed under the assumption that the sensing coverage region aligns perfectly with the communication connectivity region, and that all links are lossless and reliable.

## 6. Conclusions

In this study, to address the coverage problem in WSNs, we first introduce the 3DCIC model from the perspective of node sensing, which achieves spatial information reconstruction through inter-node collaboration. Simultaneously, we propose a jump rate-based GWO as a node scheduling strategy. Simulation experiments have been conducted to validate the dual advantages of both the 3DCIC model and the HAGWO algorithm.

Under deterministic deployment conditions, the 3DCIC model demonstrates coverage expansion factors of 2.78 times, 4.41 times, and 4.00 times compared to the binary model in regular tetrahedral, hexahedral, and octahedral deployment configurations, respectively. In 3D scenarios, the HAGWO algorithm excels in regional coverage optimization. The integration of the HAGWO algorithm with the 3DCIC model yields significant coverage gains when compared to classical binary models.

Looking forward, our research opens up several promising avenues for further investigation. First, we will extend the application of the 3DCIC model by introducing diverse obstacle types and more realistic environmental geometries to evaluate coverage performance in complex scenarios. Furthermore, to enhance the model’s adaptability and intelligence, we plan to explore a reinforcement learning framework for dynamically guiding the update strategy of the GWO algorithm, thereby optimizing its convergence and effectiveness across various environments. Another interesting direction would be to explore the discrete combinatorial analog of the proposed model, which, while formulated here in a continuous optimization framework, could facilitate different types of comparisons and theoretical analyses in future work. We will also further investigate post-deployment network performance, such as network lifetime and connectivity. Finally, analyzing the robustness of the considered models under uncertainty, such as random node failures or dynamic environmental disturbances, represents a critical and valuable area for future exploration.

## Figures and Tables

**Table 1:** Comparison of coverage gain between 3DCIC and binary models.

Deployment Mode	Magnification Times
regular tetrahedron	2.78
regular hexahedron	4.41
regular octahedron	4.00

**Table 2:** Experimental parameters for the coverage optimization problem in wireless sensor networks.

Parameter	Value
3D area size	50 m ∗ 50 m ∗ 50 m
Sensing radius Rs in 3D area	11 m
Amount of nodes in 3D area	40
Population scale	20
Max Iterations	200
RMSE Threshold ε	0.5

**Table 3:** Comparison of average moving distance.

Algorithm	Average Moving Distance (m)
HAGWO	9.041
ALGWO	9.263
GWO	9.391
IGWO	9.574
SOGWO	9.304

## Data Availability

The original contributions presented in this study are included in the article. Further inquiries can be directed to the corresponding author.
